# Effect of climatic environment on immunological features of rheumatoid arthritis

**DOI:** 10.1038/s41598-022-27153-3

**Published:** 2023-01-24

**Authors:** Yuya Kondo, Saori Abe, Hirofumi Toko, Tomoya Hirota, Hiroyuki Takahashi, Masaru Shimizu, Hisashi Noma, Hiroto Tsuboi, Isao Matsumoto, Toshiro Inaba, Takayuki Sumida

**Affiliations:** 1 Department of Rheumatology, Faculty of Medicine, University of Tsukuba, 1-1-1 Tennodai, Tsukuba, Ibaraki 305-8575 Japan; 2 Karuizawa Municipal Hospital, Karuizawa, Nagano Japan; 3 Department of Data Science, The Institute of Statistical Mathematics, Tachikawa, Tokyo Japan

**Keywords:** Rheumatology, Autoimmunity

## Abstract

The aim of this study was to clarify the effect of climatic environment on the immunological features of rheumatoid arthritis (RA). Blood samples were collected from patients with RA and healthy controls (HCs), matched by age and sex, living in two locations, Tsukuba and Karuizawa, which differ in their altitude and average air temperature and atmospheric pressure. Analysis of peripheral blood mononuclear cells (PBMCs) revealed that the proportion of T and B cell subpopulations in HCs and RA patients were significantly different between two sites. Inverse probability weighting adjustment with propensity scores was used to control for potential confounding factors. The results revealed that, in comparison with RA patients in Tsukuba, those in Karuizawa showed a significant increase in cTh1, cTfh1, and Tph cells, and significant decrease in cTh17, cTh17.1, and CD8+ Treg in T cell subpopulations, and a significant increase in DNB, DN1, DN2, and class-switched memory B cells, and a significant decrease in unswitched memory B, naïve B cells, and ABCs in B cell subpopulations. Our results suggest the possibility that climatic environment might have an effect on immune cell proportion and function, and be related to the pathogenic mechanism of RA.

## Introduction

Rheumatoid arthritis (RA) is a chronic inflammatory disorder characterized by autoimmunity, infiltration of activated inflammatory cells into the joint synovium, synovial hyperplasia, neoangiogenesis, and progressive destruction of the cartilage and bone. RA is thought to be caused by breakdown of immune tolerance, and, thus, shows some characteristic features of abnormality in acquired immune systems formed by T and B cells. Previous studies have shown that subpopulations of CD4+ T helper (Th) cells and B cells coordinately induce autoimmune synovial inflammation; Th17 cells, for example, infiltrate and induce neutrophilic inflammation in the joints, and Tfh cells not only promote autoantibody formation but also enhances their pathogenicity^1^.

It is well known that the development of RA is associated with genetic and environmental factors. Environmental risk factors include smoking and periodontal diseases mainly related with *Porphyromonas gingivalis*. These factors are hypothesized to promote aberrant citrullination and provoke local breach of tolerance to citrullinated peptides via the expression of peptidylarginine deiminase, which results in the initiation of autoimmune response and the aggravation of joint inflammation in RA^2–5^. The assumption that climatic environment influences the signs and symptoms of RA is widely believed. Indeed, anecdotally, we noticed that many patients with RA complained of fluctuation in joint symptoms according to climatic factors such as air temperature, humidity, and atmospheric pressure; however, there is a paucity of scientific evidence supporting this generally-held assumption.

Patberg et al. reviewed and evaluated evidence that suggested the signs and symptoms of RA were influenced by the weather, and revealed that temperature and humidity appeared to have effects on the symptoms of RA^6^. In addition, Savage, et al. showed that the disease activity of RA was significantly lower in both sunnier and less humid conditions^7^. Terao, et al. also reported that atmospheric pressure was inversely associated with synovitis in patients with RA, and these associations were independent from temperature and humidity^8^. Hence, the reported effects of climatic environment vary depending on the methods applied and geographic location of the studies. Moreover, it remains unclear whether climate affects the immunological pathology in patients with RA.

To elucidate the effect of climatic environment on the immunological features of RA, we collected blood samples from patients with RA and healthy controls (HC) in two distinct geographic locations: Tsukuba City, located in the flat Kanto region of Japan, at an elevation of only 33 m above sea level, and Karuizawa Town, in the mountainous prefecture of Nagano, at an average elevation of 1000 m above sea level. This contrast in altitude brings differences in average air temperature of approximately 5 °C and atmospheric pressure of approximately 100 hPa. In this study, the proportion of subpopulation in T cells and B cells were comprehensively evaluated using multi-color flow cytometry, and compared between RA patients and HCs, disease activity of RA, and by the two locations.

## Results

### Participant characteristics

A total of 80 participants were recruited for this study. Twenty patients with RA and 20 HCs were recruited from both the University of Tsukuba Hospital and from Karuizawa Municipal Hospital. The participants were matched by age and sex (Table). Among the RA patients, average of CRP and disease activity index were slightly high because a few patients were classified into moderate disease activity in patients of Tsukuba while all of the patients in Karuizawa achieved less that low disease activity. However, there were no significant differences in RF titer, positivity of anti-CCP antibody, disease activity shown by DAS28-CRP, CDAI, and SDAI, and treatment such as use or dosage of corticosteroid, methotrexate, bDMARDs, and tsDMARDs.

### Comprehensive evaluation of T cell and B cell subpopulations in RA and HCs by location

The proportion of T cell and B cell subpopulations in peripheral blood mononuclear cells (PBMCs) were comprehensively evaluated using multi-color flow cytometry, and compared between RA patients and HCs and by the two geographic locations, respectively.

**Figure 1:**
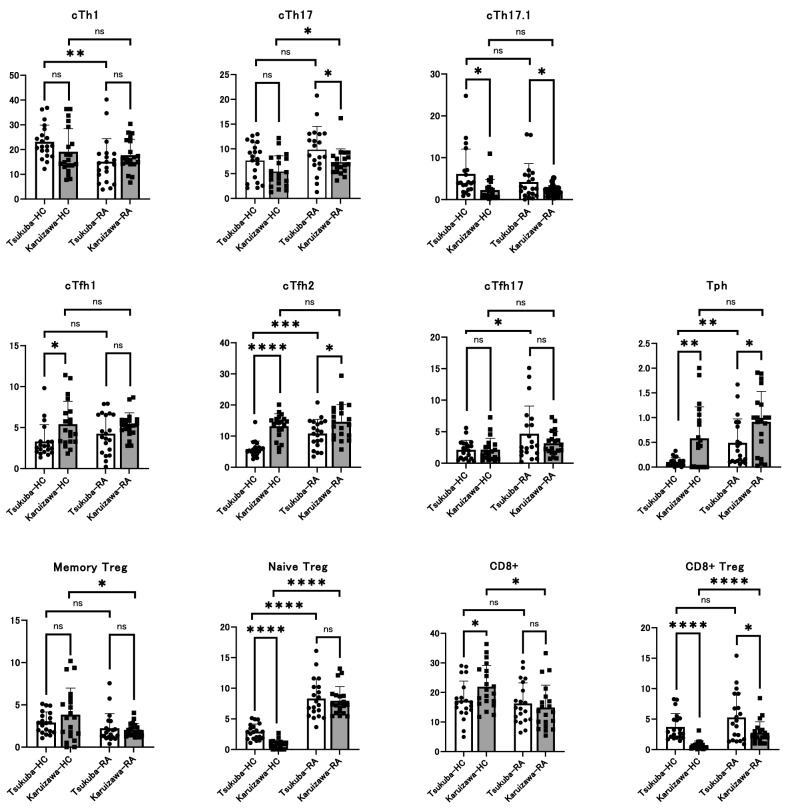
Differences in the proportion of subpopulations in peripheral blood T cells between RA patients and HCs from two distinct geographic locations. The proportion of T cell subpopulations in PBMC collected from RA patients and HCs in two hospitals was analyzed using flow cytometry. Histograms show percentage of cTh1 cells, cTh17 cells, cTh17.1 cells, cTfh1 cells, cTfh2 cells, cTfh17 cells, and cTph cells in memory CD4^+^ T cells, cTreg cells in naïve and memory CD4^+^ T cells, CD8^+^ T cells, and CD8^+^ Treg cells in CD8^+^ T cells. Data are presented as mean ± SEM, and circle and square shows samples collected from Tsukuba and Karuizawa, respectively. Unpaired t-test was performed to analyze statistical interactions between blood samples collected from HCs and RA patients in Tsukuba or Karuizawa. Statistical significance was defined as *P < 0.05, **P < 0.01, ***P < 0.001, and ****P < 0.0001.

Regarding T cells, the percentages of cTh1 cells, cTh17 cells, cTh17.1 cell, cTfh1 cells, cTfh2 cells, cTfh17 cells, and Tph cells in memory CD4^+^ T cells, Treg cells in naïve and memory CD4^+^ T cells, and CD8^+^ T cells, and CD8^+^ Treg cells in CD8^+^ T cells were analyzed. The results showed a significantly increased proportion of cTfh1 cells, memory Treg cells, and CD8+ T cells and a significantly decreased proportion of naïve Treg cells were observed only in HCs from Karuizawa compared with those from Tsukuba (Fig. 1). A significant increase in cTfh2 cells and a significant decrease in cTh17.1 cells and CD8+ Treg cells were observed in both HCs and RA patients from Karuizawa compared with those from Tsukuba (Fig. 1). Interestingly, Tph cells were significantly increased and cTh17 cells were significantly decreased only in RA patients from Karuizawa compared with those from Tsukuba (Fig. 1). Moreover, cTh17 cells, cTfh2 cells, cTfh17 cells, Tph cells, and CD8+ Treg cells were significantly increased, and cTh1 cells and memory Treg cells were significantly decreased and in patients with RA compared HCs from both Tsukuba and Karuizawa, and there was no difference between Tsukuba and Karuizawa regarding the fluctuations of the proportions of these cells (Fig. 1). A significant increased proportion of naïve Treg cells was observed in patients with RA compared to HCs from both Tsukuba and Karuizawa (Fig. 1). Collectively, there were some significant differences between Tsukuba and Karuizawa regarding T cell subpopulations, whereas their fluctuations between HCs and RA patients were comparable between Tsukuba and Karuizawa.

**Figure 2:**
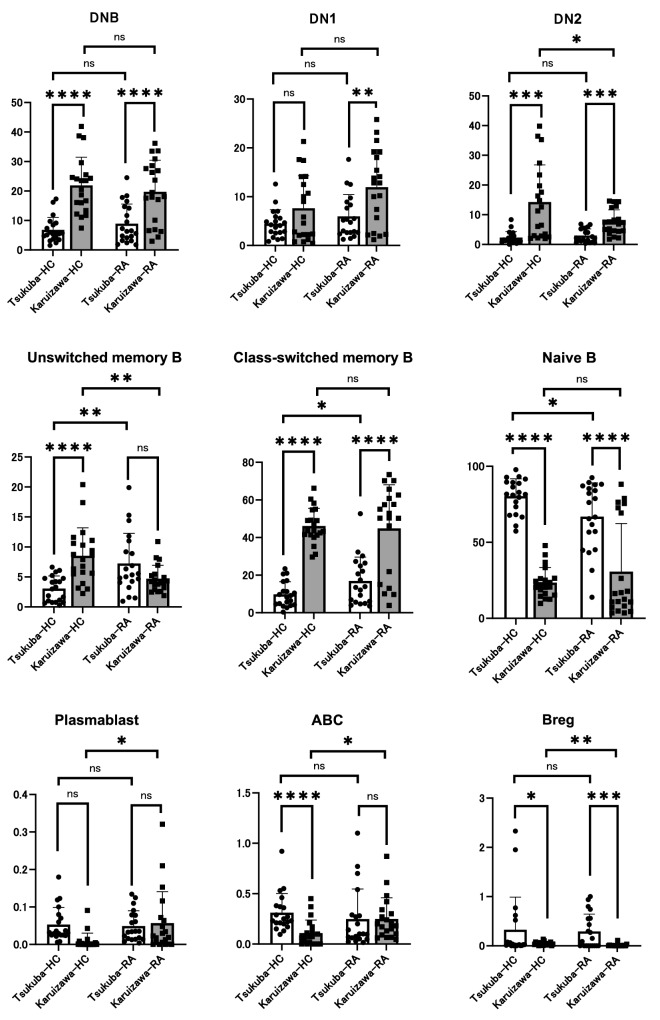
Differences in the proportion of subpopulations of peripheral blood B cells between RA patients and HCs from two distinct geographic locations. The proportion of subpopulation in B cells in PBMC collected from RA patients and HC in two hospitals was analyzed by flow cytometry. Histograms show percentage of class switched memory B cells, unswitched memory B cells, memory B cells, and double negative (DN) B cells in CD19^+^CD20^+^ cells, DN1 cells and DN2 cells in DNB cells, plasmablast in CD19^+^CD20^−^ cells, ABC and Breg cells in CD19^+^ cells. Data are presented as mean ± SEM. Unpaired t-test was performed to analyze a statistical interaction between blood samples collected from HC or RA patients in Tsukuba or Karuizawa. Statistical significance was defined as *P < 0.05, **P < 0.01, ***P < 0.001, and ****P < 0.0001.

Regarding B cells, the percentage of double negative (DN) B cells, DN1 cells, DN2 cell, naïve B cells, unswitched memory B cells, class switched B cells, and plasmablasts in CD19+CD20+ B cells, and B regulatory (Breg) cells in CD19^+^ B cells was analyzed. A significant increased proportion of unswitched memory B cells and significant decreased proportion of ABCs were observed in the HCs from Karuizawa compared to those from Tsukuba (Fig. 2). A significant increase in DNB cells, DN2 B cells, and class-switched memory B cells and a significant decrease in naïve B cells and Breg cells were observed in both HCs and RA patients from Karuizawa compared with those from Tsukuba (Fig. 2). Moreover, DN2 B cells were significantly increased in RA patients from Karuizawa compared with those in Tsukuba (Fig. 2). Unswitched memory B cells and class switched memory B cells were significantly increased, and naïve B cells were significantly decreased in RA patients compared to HCs in Tsukuba, while plasmablast and ABC were significantly increased, and unswitched memory B cells, DN2 cells, and Breg cells were significantly decreased in RA patients compared to HCs in Karuizawa (Fig. 2). Interestingly, our data showed discordance between Tsukuba and Karuizawa regarding the fluctuations in the proportions of these cells, especially in unswitched memory B cells (Fig. 2). Collectively, comparing Tsukuba and Karuizawa, there were some significant differences and inconsistencies regarding B cell subpopulations of HCs and/or RA patients. Accordingly, the proportion of the subpopulations of T cells and B cells were significantly different between Tsukuba and Karuizawa, and some differences were only observed in the patients with RA, indicating that the climate variations might affect immune function in both the normal and pathogenic mechanisms of RA.

### Comparison of T and B cell subpopulations in the patients with RA and HCs after propensity score weighting

Although there were no significant differences in baseline characteristics of RA patients and HCs between Tsukuba and Karuizawa (Table 1), inverse probability weighting (IPW) adjustments with propensity scores were performed to control for any bias caused by the imbalance of potential confounding factors.

**Table 1:** Demographics and clinical characteristics of HCs and RA patients.

	University of Tsukuba Hospital		Karuizawa Municipal Hospital	
	HC (N = 20)	RA (N = 20)	HC (N = 20)	RA (N = 20)
Age	58 ± 12	62 ± 12	60 ± 12	65 ± 11
Female, n (%)	20 (100)	18 (90)	15 (75)	16 (80)
CRP (mg/dL)	–	0.62 ± 1.43	–	0.29 ± 0.53
RF (U/ml)	–	55 ± 63	–	71 ± 126
Anti-CCP antibody, n (%)	–	15 (88^a^)	–	14 (70^a^)
DAS28-CRP	–	2.00 ± 0.76	–	1.79 ± 0.84
CDAI	–	4.3 ± 3.5	–	2.9 ± 2.5
SDAI	–	4.6 ± 3.6	–	3.2 ± 2.8
PSL use, n (%)	–	6 (30)	–	8 (40)
PSL dose, (mg/day)	–	4.1 ± 2.8	–	3.6 ± 1.1
MTX use, n (%)	–	14 (70)	–	17 (85)
MTX dose, (mg/week)		7.4 ± 3.2	–	8.0 ± 4.0
b/tsDMARDs use, n (%)	–	6 (30)	–	4 (20)

Anti-CCP antibody, anti-cyclic citrullinated peptide antibody; b/tsDMARDs, biologic or targeted synthesized disease modifying anti-rheumatic drugs; CDAI, Clinical Disease Activity Index; CRP, C-reactive protein; MTX, methotrexate; PSL, prednisolone; RF, rheumatoid factor; SDAI, Simplified Disease Activity Index. Continuous variables are showed by Mean ± standard deviation (SD).

^a^Anti-CCP antibody was measured in 20 RA patients from the University of Tsukuba Hospital and Karuizawa Municipal Hospital, respectively (N = 40).

**Figure 3:**
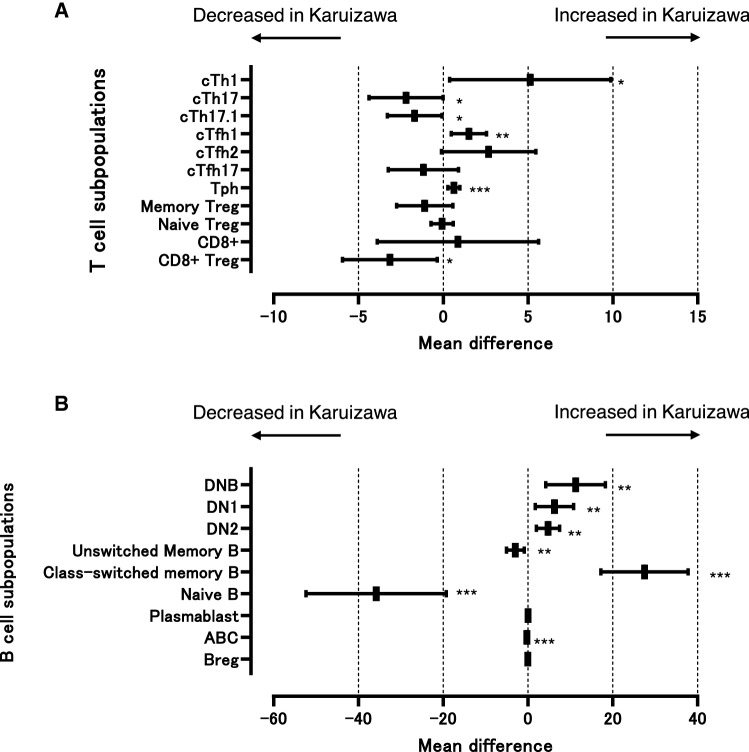
Comparison of subpopulations of peripheral blood T and B cells in RA patients after adjustment with propensity score weighting. Inverse probability weighting adjustments with propensity scores were performed to control for biases caused by the imbalance of potential confounding factors in comparison of subpopulation in T cells (**A**) and B cells (**B**) of PBMC collected from RA patients in two hospitals. The graph shows the mean difference with 95% confidence intervals for each subpopulation after IPW adjustment. Positive or negative numbers represent increase or decrease of the subpopulations in the patients in Karuizawa, respectively. Statistical significance was defined as *P < 0.05, **P < 0.01, ***P < 0.001, and ****P < 0.0001.

In T cell subpopulations, a significant increase in cTh1 cells, cTfh1 cells, and Tph cells, and a significant decrease in cTh17 cells, cTh17.1 cells, and CD8+ Treg cells were observed in the Karuizawa patients with RA compared those from Tsukuba after the IPW adjustments (Fig. 3A). In addition, analyses of B cell subpopulations showed that DNB cells, DN1 B cells, DN2 B cells, and class-switched memory B cells were significantly increased, and unswitched memory B cells, naïve B cells, and ABCs were significantly decreased in the Karuizawa patients with RA compared with those from Tsukuba (Fig. 3B). Accordingly, the differences in T and B cell subpopulations between Karuizawa and Tsukuba RA patients were confirmed even after IPW adjustment.

**Figure 4:**
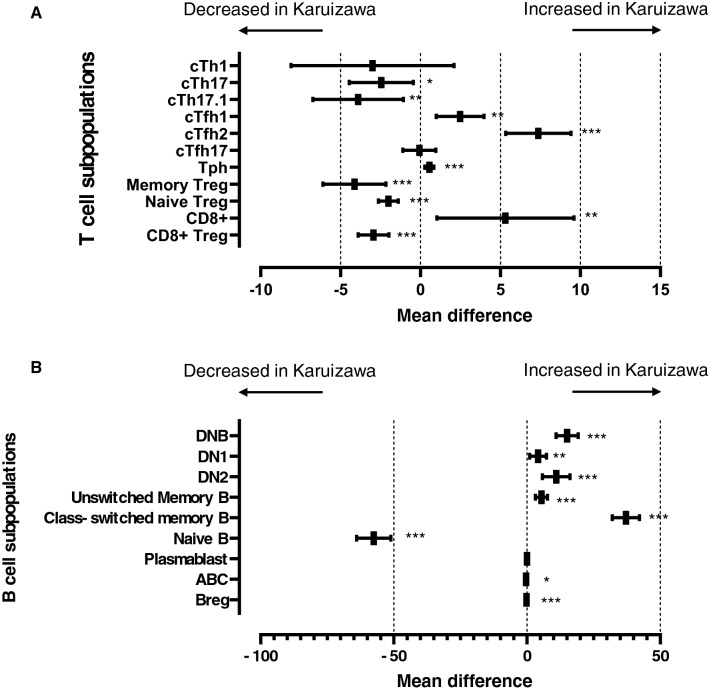
Comparison of subpopulations of peripheral blood T and B cells in HCs after adjustment with propensity score weighting. Inverse probability weighting adjustments with propensity scores were performed to control for biases caused by the imbalance of potential confounding factors in comparison of subpopulation in T cells (**A**) and B cells (**B**) of PBMC collected from HCs in two hospitals. The graph shows the mean difference with 95% confidence intervals for each subpopulation after IPW adjustment. Positive or negative numbers represent increase or decrease of the subpopulations in the patients in Karuizawa, respectively. Statistical significance was defined as *P < 0.05, **P < 0.01, ***P < 0.001, and ****P < 0.0001.

IPW adjustments in HCs revealed that a significant increase in cTfh1 cells, cTfh2 cells, Tph cells, and CD8+ T cells, and a significant decrease in cTh17 cells, cTh17.1 cells, memory and naïve Treg cells, and CD8+ Treg cells in the Karuizawa patients with RA compared those from Tsukuba (Fig. 4A). Moreover, analyses of B cell subpopulations showed that DNB cells, DN1 B cells, DN2 B cells, and unswitched and class-switched memory B cells were significantly increased, and naïve B cells, ABCs, and Breg cells were significantly decreased in the Karuizawa patients with RA compared with those from Tsukuba (Fig. 4B). These observations showed that the proportion of the subpopulations of T cells and B cells were significantly different in both RA patients and HCs between Karuizawa and Tsukuba after the control for the imbalance of potential confounding factors with IPW adjustment, and suggesting that the climate variations might essentially affect immune cell phenotype regardless of with or without RA.

### Correlation between disease activity of RA and subpopulations of T cells and B cells

**Figure 5:**
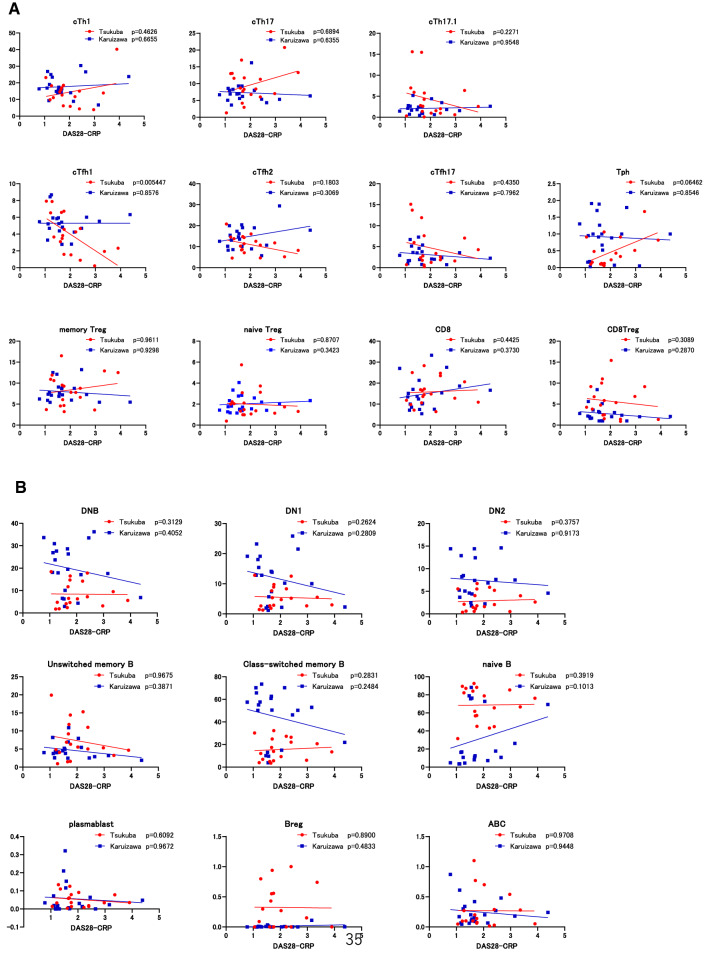
Correlation between T cell or B cell subpopulations and disease activity in RA patients. Correlation between disease activity of RA and proportion of the subpopulations in T cells (**A**) and B cells (**B**) in PBMCs collected from Tsukuba and Karuizawa were analyzed. Correlation analysis was performed using Spearman rank correlation coefficient.

The relationship between disease activity of RA and subpopulation in T cells and B cells, respectively, were assessed. As described above, there were no significant difference in disease activity of RA between Tsukuba and Karuizawa, and most of patients achieved low disease activity (Table 1 and Fig. 5). Among T cell subpopulations, the proportion of cTfh1 cells negatively correlated with DAS28-CRP in Tsukuba but not in Karuizawa (Fig. 5A). Proportion of Tph cells also tended to be positively correlated with DAS28-CRP only in Tsukuba (Fig. 5A). On the other hand, no significant correlations were observed between disease activity of RA and subpopulations in B cells for both Tsukuba and Karuizawa (Fig. 5B). Taken together, these findings suggest that the disease activity of RA might have less of an effect on T and B cell subpopulations in patients with RA achieving low disease activity regardless of climate.

## Discussion

In this study, we aimed to comprehensively evaluate the proportions of CD4+ T cell and B cell subpopulations in peripheral blood collected from HCs and RA patients, and compared them between two locations; Tsukuba City and Karuizawa Town, which differ in altitude by 1000 m and, thus, have distinct differences in average air temperature and atmospheric pressure. Studying populations from these two locations, therefore, allows us to elucidate the effect of climate on the immunological features of RA. IPW adjustment with propensity scores was adopted to control for potential confounding factors such as age, sex, positivity of RF and anti-CCP antibody, dose of prednisolone, dose of methotrexate, use of bDMARDs or tsDMARDs, and DAS28-CRP in the RA cohorts, and age and sex in the HC cohorts between from Tsukuba and Karuizawa. Our analysis revealed that the proportion of T and B cells subpopulations were significantly different not only in RA patients but HCs between Tsukuba and Karuizawa, suggesting that climate variations might essentially affect immune cell phenotype regardless of background characteristics, like with or without RA. In addition, some of those differences of T and B cell subpopulations were observed only in the patients with RA, indicating that they might be a characteristic immunophenotype in RA.

In T cell subpopulations, a significant increase in cTh1 cells, cTfh1 cells, and Tph cells and significant decrease in cTh17 cells, cTh17.1 cells, and CD8+ Treg cells was observed in the patients with RA from Karuizawa compared with those of Tsukuba after IPW adjustment. Among these T cell subpopulations, Tph cells tended to be increased, and cTh17 cells and CD8+ Treg cells were significantly increased in RA patients compared to HCs from Karuizawa. However, there were no significant correlations between disease activity of RA and the T cell subpopulations in which a significant difference was found when Tsukuba and Karuizawa were compared. IFNγ secreting Th1 cell was identified in synovial fluids from RA patients^9,10^, and induce macrophage activation characterized by an increased capacity to produce pro-inflammatory cytokines such as TNF^11^, Past study reported that there was no significant difference in peripheral blood CXCR3+CCR6− Tfh1 cell between RA patients and HCs^12^. Tph cells have been defined as PD-1^hi^CXCR5^-^CD4^+^ T cells, and reported to be uniquely poised to promote B cell response and antibody production within pathologically inflamed non-lymphoid tissue in RA^13^. Combining mass cytometry and transcriptomics also revealed expansion of Tph cells in RA synovia^14^. Our previous study and other reports have indicated the pathogenetic role of Th17 cells in RA^15–17^. Th17.1 cells are a subgroup of Th17 cell characterized by the expression of CXCR3 and the production of IFNγ, and have been reported as the most pathogenic among the Th17 cells and as the predictor of therapeutic response in patients with RA^18,19^. CD122^+^CD8^+^Treg cells have the capacity to inhibit T cell responses and suppress autoimmunity, however, their role in RA has remained unclear^20^. Hence, it has been speculated that climatic environment might affect the pathology of RA through alternation of T cell subpopulations.

Analyses of B cell subpopulations showed that, after IPW adjustment, DNB cells, DN1 B cells, DN2 B cells, and class-switched memory B cells were significantly increased, and unswitched memory B cells, naïve B cells, and ABCs were significantly decreased in the patients with RA from Karuizawa compared with those from Tsukuba. Among these B cell subpopulations, unswitched memory B cells were significantly decreased, but ABCs were significantly increased in RA patients compared to HCs from Karuizawa. However, there was no significant correlation between disease activity of RA and the B cell subpopulations in which significant difference was found when Tsukuba and Karuizawa were compared. DNB cells have been defined as IgD-CD27- B cells and are subclassified into DN1 cells or DN2 cells according to expression of CXCR5. DNB cells have garnered interest in the field of autoimmunity, especially in systemic lupus erythematosus (SLE); autoreactive DN2 B cells were expanded and differentiated into autoantibody-secreting plasmablast via hyper-responsiveness to Toll-like receptor 7 in extra-follicle^21^. With regards RA, several studies have reported that DNB cells were increased in RA, particularly in ACPA+ patients^22,23^. On the other hand, immunoglobulin class switching and further differentiation of memory B cells were mediated by T-B interaction in the germinal center, and the enhancement of this process was suggested by two findings: firstly, that citrullinated antigen-specific B cells displayed markers of class-switched memory B cells^24^ and, secondly, that the number of class-switched memory B cells was significantly increased in subjects carrying the risk haplotype B lymphoid kinase (BLK), which is a member of the Src family of tyrosine kinases and associated with RA^25,26^. ABC was newly identified B cells subset, and found to accumulate in the spleens of aged mouse and model mice of systemic lupus erythematosus^27,28^. Furthermore, the expansion of human ABCs has been observed in many autoimmune diseases including RA^29^. Accordingly, it was conjectured that climatic environment might also affect the pathology of RA through alternation of B cell subpopulations.

Although our analysis revealed some significant altered proportion of T and B cell subpopulations when comparing Tsukuba and Karuizawa populations, it is unclear how these cell alterations interact reciprocally and regulate the pathology of RA. As mentioned above, it was reported that Tph cells play an important role in promotion of B cell response and antibody production^13,14^, and that DNB cells and class-switched memory B cells are also related with autoantibody formation including ACPA formation in RA^22–24^. Consequently, increase of Tph cells, DNB cells, and class-switched memory B cells in the Karuizawa population raises the possibility that enhancement of autoantibody production might be one of the underlying mechanisms of RA related to climatic environment.

The question remains as to how the climatic factors such as air temperature and air pressure regulate the differentiation and the function of immune cells in RA. Significant relationships have been reported regarding the number and percentage of CD4+, CD8+ T cells, CD20+ B cells, and ambient temperature, sunlight duration, and air pressure in healthy volunteers^30^. The systematic effect of general cooling by 5-min exposure to cold air at a temperature of − 25 °C in healthy volunteers leads to decrease of T-lymphocytes count in venous blood, which indicated their functional insufficiency^31^. Although it has been reported that environmental factors such as oxygen concentration^32,33^, acidification^34,35^, salt concentration^36^, and glucose, amino acid, and lipid metabolism^37–39^ altered the differentiation and the function of immune cells, and contributed to the pathology of autoimmune diseases, it remains unclear how climatic factors such as air temperature and air pressure regulate immune cell function and the development of autoimmune diseases including RA.

The current study has some limitations. First, the effect of air temperature on the results of our study was inferred to be slight, because air temperature is almost completely controlled in the average Japanese living environment. Second, patients recruited for this study, from both Tsukuba and Karuizawa, were undergoing treatment and their RA was well-controlled with anti-rheumatic therapies including b/tsDMARDs, which may have substantially affected and modified the results of our study. Indeed, our results in HCs revealed that the difference in proportion of peripheral blood immune cells seemed to be more remarkable, and observed in more T and B cell subpopulations than RA patients. Third, as mentioned above, we were not able to clarify how climatic environment regulates immune cell function and disease state. Forth, it was required to freezing all blood samples for preservation. In addition, samples in Karuizawa were needed to be transported to Tsukuba to be analyzed in Tsukuba. The results of FACS were not statistical but slightly different in some immune cell subsets such as cTh17, cTfh17, and Breg cells (Supplement Figs. 3 and 4) between with and without freezing preservation, and thus it seemed to be difficult to completely exclude the possibility of the effect of freezing preservation and transportation on our results. Further studies that include more patients with high disease activity or without therapeutic intervention are needed to elucidate the exact and specific mechanism how climatic environment affects the immune cell-mediated pathology of RA.

In conclusion, our results suggest the possibility that climatic environment such as air temperature and air pressure has an effect on the proportion of T and B cell subpopulations and their function, and is related to the pathogenic mechanism of RA including autoantibody formation induced by T-B interaction.

## Material and methods

### Study participants

In this study, patients with RA and HCs were recruited from the University of Tsukuba Hospital and Karuizawa Municipal Hospital, respectively. Blood samples were collected from 20 RA patients receiving treatment in the Department of Rheumatology and a further 20 samples from HC volunteers were provided by Tsukuba Human-Tissue Biobank Center in the University of Tsukuba Hospital. Likewise, blood samples were collected from 20 patients with RA and 20 HC volunteers in Karuizawa Municipal Hospital. Although we collected blood samples without restriction of the time of year, there was no seasonal bias in both Karuizawa and Tsukuba, and thus the effect of time of sample collection was considered to be a minimum. All patients with RA fulfilled either the 1987 revised criteria of the American College of Rheumatology (ACR) for the classification of RA or the 2010 ACR/European League Against Rheumatism (EULAR) classification criteria. All RA patients were evaluated for age, sex, tender joint count (TJC), swollen joint count (SJC), patient global assessment (patient visual analogue scale [Pt-VAS]), physician global assessment (doctor’s visual analogue scale [D-VAS]), C-reactive protein (CRP) level, and disease activity index of RA such as Disease Activity Score 28 (DAS28) -CRP, Clinical Disease Activity Index (CDAI), and Simplified Disease Activity Index (SDAI) were calculated based on above data. Rheumatoid factor (RF) value, positivity of anti-cyclic citrullinated peptide (CCP) antibody, and medication at the time when the blood samples were collected were assessed using electronic medical records.

The study was approved by the ethics committees of the University of Tsukuba Hospital and was carried out in accordance with the Declaration of Helsinki. Patients recruited for the study were enrolled after written informed consent was received (the reference number: H30-134).

### Cell preparation

After obtaining whole blood samples, collected using heparin tubes, peripheral blood mononuclear cells (PBMCs) were isolated by density gradient using Ficol-Paque Plus (GE Healthcare). To evaluate several samples simultaneously, PBMCs were cryopreserved in CELLBANKER (TakaraBio) at − 80 °C and stored in a deep freezer. The process for preserving blood samples was standardized and not different between Karuizawa and Tsukuba. Frozen samples of Karuizawa were transported to Tsukuba, and all of the samples were analyzed in Tsukuba University Hospital. Before evaluation, PBMCs were thawed, rested for at least 1 h to allow removal of cell debris as recommended in the protocol (catalog 3520-2A, MABTECH), washed, and resuspended in RPMI Medium 1640 containing 10% FBS and 1% penicillin.

### Flow cytometry analysis

Before superficial antigen staining, cells were stained with 7-Amino-Actinomycin D (7-AAD) for 5 min at room temperature for the exclusion of nonviable cells in the flow cytometry analysis. Surface staining of the subpopulations in T and B cells was conducted for 30 min on ice under darkened conditions with the following antibodies for the analysis of T cells: anti-CD4-APC (BioLegend), anti-CD8-APC-Alexa700 (BIoLegend), anti-CD25-BV711 (BioLegend), anti-CD45RA-APC-Cy7 (BD), anti-CD122-BV421 (BioLegend), anti-CD127-BV605 (BioLegend), anti-CC chemokine receptor 6 (CCR6)-PE (BioLegend), anti-CXC chemokine receptor 3 (CXCR3)-Alexa Fluor (AF) 488 (BioLegend), anti-CXCR5-PE-Cv7 (BioLegend), and anti-PD-1-BV510 (BioLegend); and for the analysis of B cell: anti-CD11c-BV711 (BioLegend), anti-CD19-FITC (BioLegend), anti-CD20-APC-Cy7 (BioLegend), anti-CD24-BV510 (BioLegend), anti-CD27-APC (BioLegend), anti-CD38-PE-Cy7 (BioLegend), anti-CXCR5-Pacific Blue (BioLegend), and anti-IgD-PE (BioLegend). FACS analysis was performed using LSRFortessa X-20 Flow Cytometer (BD Bioscience), and analyzed with FlowJo software (Tree Star, Ashland, OR, USA).

In this study, the subpopulations of T cells and B cells were defined using cell surface markers reported in previous studies. Definitions of each subpopulation are summarized in the Supplemental Table. Representative plots and the gating strategy for evaluating T cells and B cells are shown in Supplement Figs. 1 and 2. We also confirmed that there was no difference of the results of FACS analysis of blood sample with or without freezing preservation by exclusion of nonviable cells stained with 7-AAD. Results are shown in Supplement Figs. 3 and 4.

### Statistical analysis

Data are summarized as mean ± standard deviation (SD). Statistical differences in baseline characteristics were evaluated using the Mann–Whitney U test or the Kruskal–Wallis test for continuous variables and by Fisher’s exact test or Chi-squared test for categorical variables. Correlation analysis was performed using Spearman’s rank correlation coefficient.

Student’s t-tests were performed to assess the differences between blood samples collected from HCs or RA patients in Tsukuba and Karuizawa. Wilcoxon signed rank tests were performed to assess the differences between blood samples before and after freezing preservation. It was anticipated that the background characteristics of RA patients and HCs would differ substantially between the Tsukuba and Karuizawa groups, thus, inverse probability weighting (IPW) adjustments with propensity scores were performed to control for biases caused by potential confounding factors. We considered the following variables as potential confounding factors: age, sex, positivity of RF and anti-CCP antibody, dose of prednisolone, dose of methotrexate, use of biologic disease-modifying anti-rheumatic drugs (bDMARDs) or targeted disease-modifying anti-rheumatic drugs (tsDMARDs), and DAS28-CRP in RA patients, and age and sex in HCs. We used the multiple imputation by chained equation^40^ to treat missing data. We created 200 imputation data and used ordinary Rubin’s synthesis rule. We used the R package mice^41^ for the computation. Since this study is an observational study, and there are no families of statistical tests that multiplicity adjustments should be considered^42^. We did not adopt multiplicity adjustments for all statistical tests. All *P* values quoted are 2-sided and the significant levels were set to 0.05.

## Supplementary Information

Supplementary Information.

## Data Availability

The datasets generated during and/or analyzed during the current study are available from the corresponding author on reasonable request.
