# Supplementary material for: Effect of climatic environment on immunological features of rheumatoid arthritis
Source: Sci Rep. 2023 Jan 24;13:1304. doi: 10.1038/s41598-022-27153-3 (PMC9873807; doi:10.1038/s41598-022-27153-3)

Supplemental Information

Supplement Figure 1. Gating strategy of T cell subpopulations

Representative plots and gating strategy for evaluating the T cell subpopulations are shown.

Supplement Figure 2. Gating strategy of B cell subpopulations

Representative plots and gating strategy for evaluating the B cell subpopulations are shown.

Supplement Figure 3. Comparison of T cell subpopulations before and after freezing blood samples

The proportion of T cell subpopulations in PBMC collected from HCs was compared between before and after freezing the samples. Graphs show percentage of cTh1 cells, cTh17 cells, cTh17.1 cells, cTfh1 cells, cTfh2 cells, cTfh17 cells, and cTph cells in memory CD4$^+$ T cells, cTreg cells in naïve and memory CD4$^+$ T cells, CD8$^+$ T cells, and CD8$^+$ Treg cells in CD8$^+$ T cells. Open and closed circles and dashed line between them show freshly prepared and cryopreserved samples collected from each individual subject. Wilcoxon signed rank test was performed to analyze statistical differences between the samples before and after freezing preservation.

Supplement Figure 4. Comparison of B cell subpopulations before and after freezing blood

samples

The proportion of B cell subpopulations in PBMC collected from HCs was compared between before and after freezing the samples. Graphs show percentage of class switched memory B cells, unswitched memory B cells, memory B cells, and double negative (DN) B cells in $CD19^+CD20^+$ cells, DN1 cells and DN2 cells in DNB cells, plasmablast in $CD19^+CD20^-$ cells, ABC and Breg cells in $CD19^+$ cells. Open and closed circles and dashed line between them show freshly prepared and cryopreserved samples collected from each individual subject. Wilcoxon signed rank test was performed to analyze statistical differences between the samples before and after freezing preservation.

Supplement Table. Definition of subpopulation of T and B cells.

T cell	Patterns of cell surface marker
$CD4^+$ T cell	$CD4^+CD8^-$
cTh1 cell	$CD4^+CD8^-45RA^-CXCR5^-CXCR3^+CCR6^-$
cTh2 cell	$CD4^+CD8^-45RA^-CXCR5^-CXCR3^-CCR6^-$
cTh17 cell	$CD4^+CD8^-45RA^-CXCR5^-CXCR3^-CCR6^+$
cTh17.1 cell	$CD4^+CD8^-45RA^-CXCR5^-CXCR3^+CCR6^+$
cTfh cell	$CD4^+CD8^-45RA^-CXCR5^+$
cTfh1 cell	$CD4^+CD8^-45RA^-CXCR5^+CXCR3^+CCR6^-$
cTfh2 cell	$CD4^+CD8^-45RA^-CXCR5^+CXCR3^-CCR6^-$
cTfh17 cell	$CD4^+CD8^-45RA^-CXCR5^+CXCR3^-CCR6^+$
cTfh17.1 cell	$CD4^+CD8^-45RA^-CXCR5^-CXCR3^+CCR6^+$
cTph cell	$CD4^+CD8^-45RA^-CXCR5^-PD\text{-}1^+$
cTreg cell	$CD4^+CD8^-CD45RA^-CD25^+CD127^-$
$CD8^+$ T cell	$CD4^-CD8^+$
$CD8^+$ Treg cell	$CD4^-CD8^+CD122^+$
**B cell**	**Patterns of cell surface marker**
Naïve B cell	$CD19^+CD20^+CD27^-IgD^+$

Unswitched memory B cell	$CD19^{+}CD20^{+}CD27^{+}IgD^{+}$
Class switched memory B cell	$CD19^{+}CD20^{+}CD27^{+}IgD^{-}$
Double negative B cell	$CD19^{+}CD20^{+}CD27^{-}IgD^{-}$
Double negative B1 cell	$CD19^{+}CD20^{+}CD27^{-}IgD^{-}CXCR5^{+}$
Double negative B2 cell	$CD19^{+}CD20^{+}CD27^{-}IgD^{+}CXCR5^{-}$
Plasmablast	$CD19^{+}CD20^{-}CD27^{+}CD38^{+}$
ABC	$CD19^{+}CD11c^{+}$
Breg	$CD19^{+}CD24^{+}CD27^{+}$

c, circulating; Th, T helper; Tfh, T follicular helper; Treg, T regulatory; CCR, CC chemokine receptor, CXCR, CXC chemokine receptor; PD-1, programmed cell death 1; ABC, age-associated B cell; Breg, B regulatory.

Supplement Figure 1

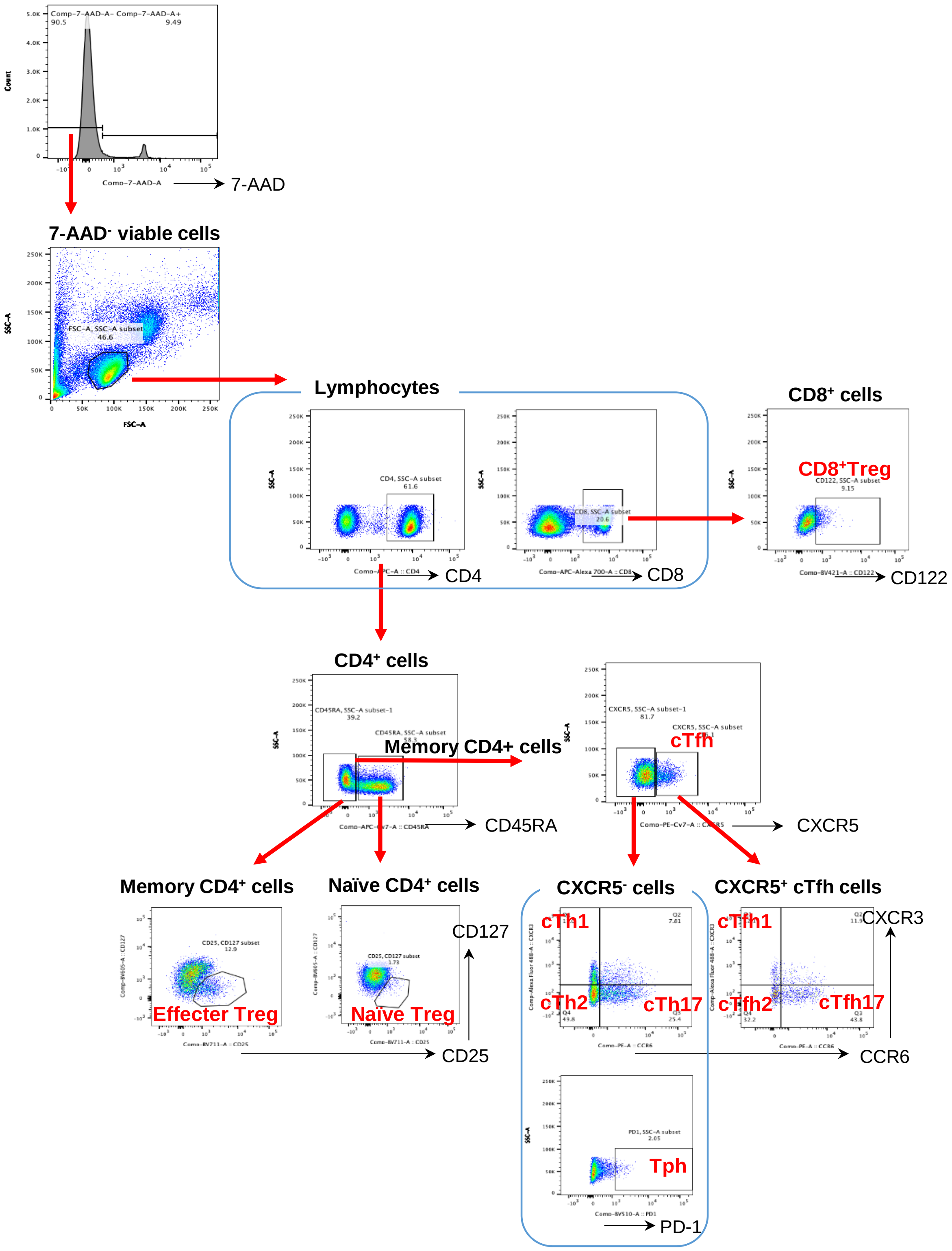

Supplement Figure 2

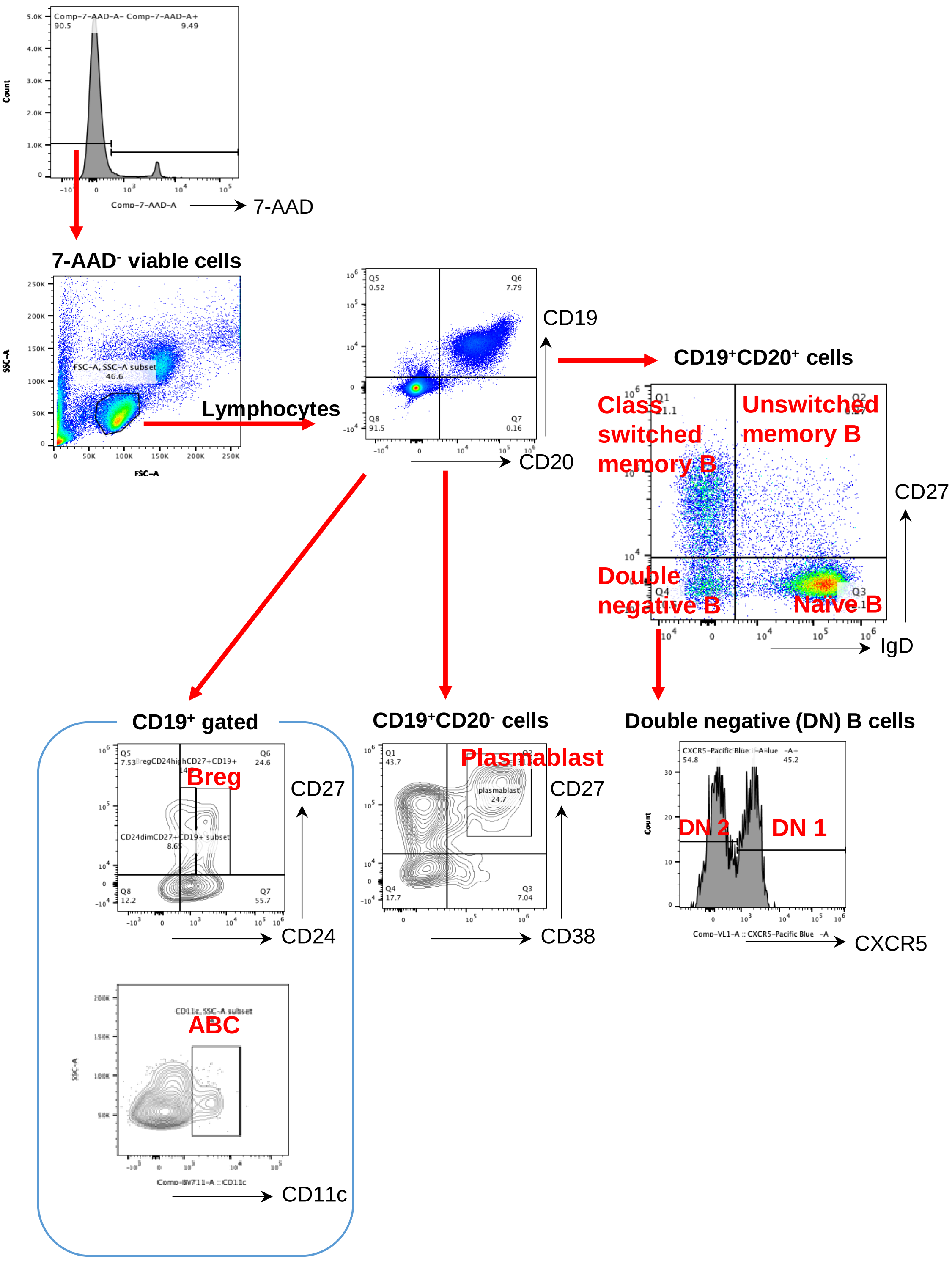

Supplement Figure 3

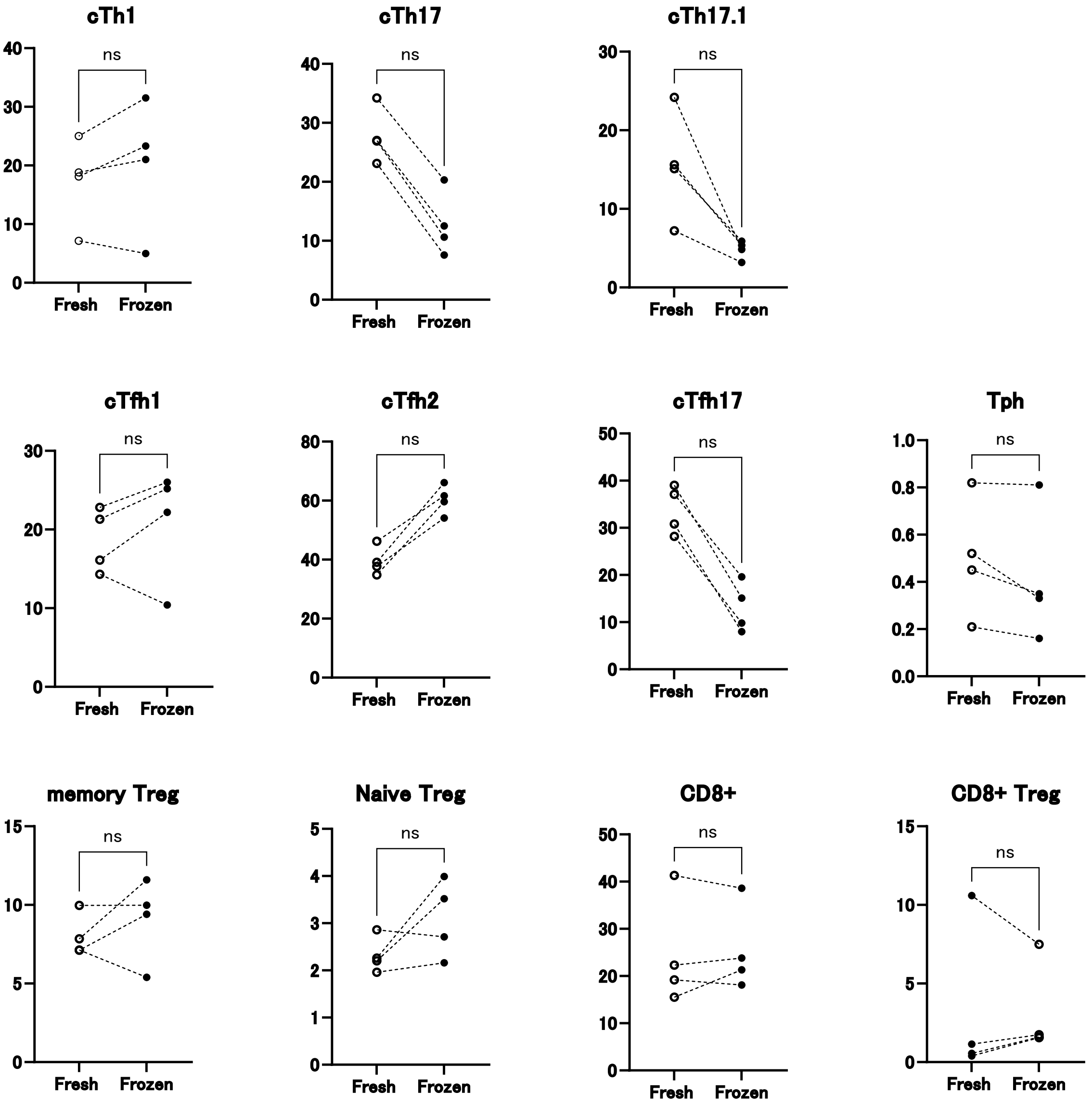

Supplement Figure 4

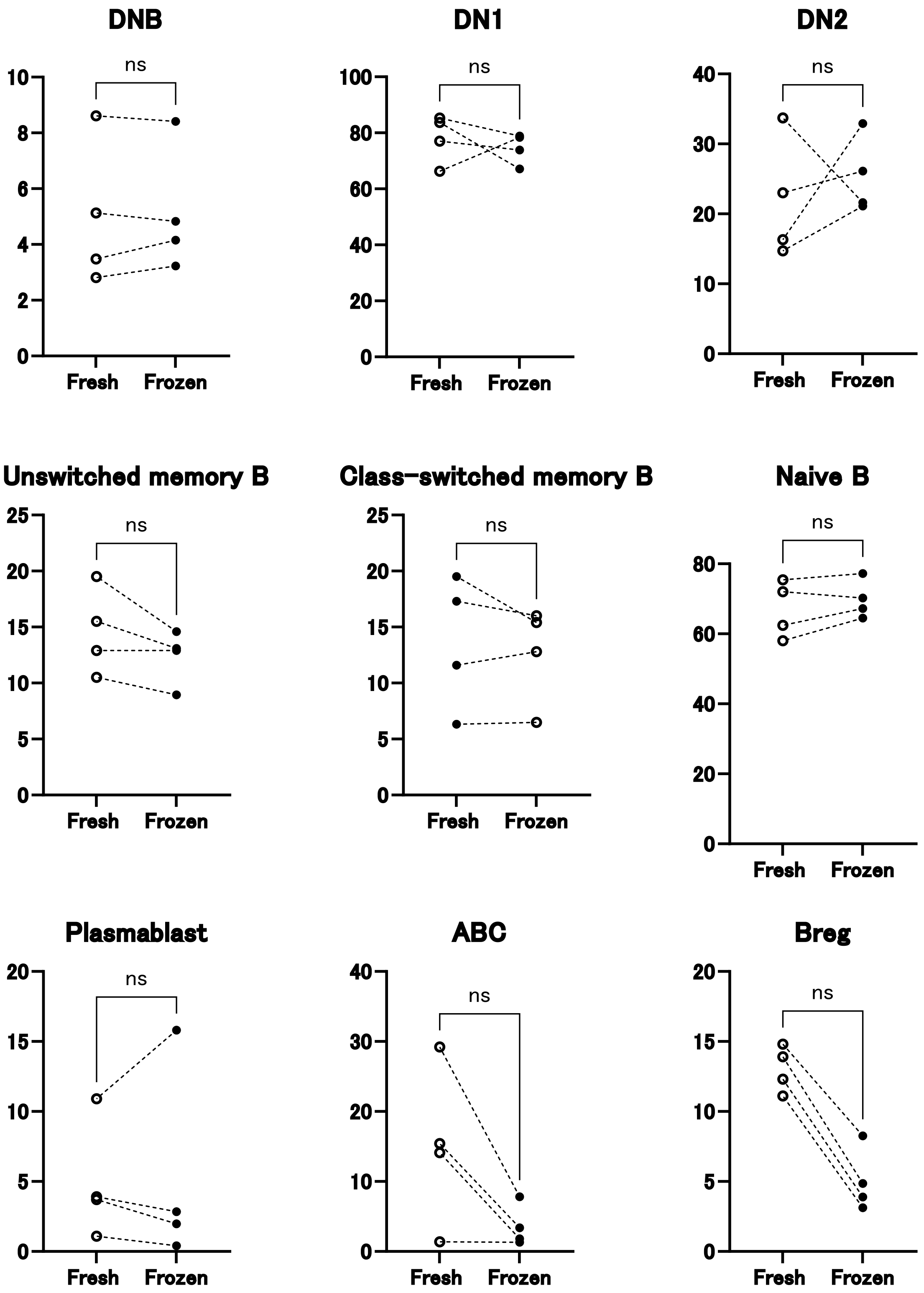

Supplement: Supplementary file 1 — Supplementary Information. [file 41598_2022_27153_MOESM1_ESM.pdf]
